# Characterising the Smoking Status and Quit Smoking Behaviour of Aboriginal Health Workers in South Australia

**DOI:** 10.3390/ijerph10127193

**Published:** 2013-12-13

**Authors:** Lauren Maksimovic, Catherine Paquet, Mark Daniel, Harold Stewart, Alwin Chong, Peter Lekkas, Margaret Cargo

**Affiliations:** 1 Cancer Council South Australia, P.O. Box 929, Unley BC, South Australia 5061, Australia; E-Mails: lmaksimovic@cancersa.org.au (L.M.); hstewart@cancersa.org.au (H.S.); 2 Social Epidemiology and Evaluation Research Group, School of Population Health, University of South Australia, City East Campus, North Terrace, G.P.O. Box 2471, CEA-01, Adelaide, South Australia 5001, Australia; E-Mails: catherine.paquet@unisa.edu.au (C.P.); mark.daniel@unisa.edu.au (M.D.); peter.lekkas@unisa.edu.au (P.L.); 3 Aboriginal Health Council SA, P.O. Box 981, Unley, South Australia 5061, Australia; E-Mail: alwin.chong@adelaide.edu.au

**Keywords:** smoking cessation, Aboriginal health workers, tobacco control, participatory research

## Abstract

The study objectives were to characterise the smoking status and quit smoking behaviour of Aboriginal Health Workers (AHWs) in South Australia (SA), Australia; and identify the psychosocial, socio-demographic, and household smoking characteristics that distinguish smokers from quitters and never smokers. A self-reported cross-sectional survey was completed by AHWs in SA. Non-parametric statistics were used for inferential analyses. Eighty-five AHWs completed surveys representing a response rate of 63.0%. The prevalence of current smokers was 50.6%. Non-smokers (49.5%) included quitters (22.4%) and never smokers (27.1%). Smoking status did not differ by gender or geographic location. Of current smokers, 69.0% demonstrated a readiness to quit and 50.0% had made at least one quit attempt in the last 12 months. Compared to quitters and never smokers, current smokers expressed lower emotional wellbeing, and three times as many resided with another smoker. Quitters had the highest levels of perceived social support and part-time employment. A high proportion of AHWs who smoke desire, and are ready to quit. Individual, social and household factors differentiated smokers from non-smokers and quitters. Social support, and relationships and structures that favour social support, are implicated as necessary to enable AHWs who smoke to act on their desire to quit smoking.

## 1. Introduction

The prevalence of tobacco smoking for Australia’s Aboriginal and Torres Strait Islander people is 46.8% [1], an estimate more than double that of the general Australian population (19%) [2]. Moreover, smoking-associated morbidity and mortality accounts for 17% of the health gap between Aboriginal and Torres Strait Islander people and the total Australian population [3]. Reducing the prevalence of smoking among Aboriginal people has been identified as integral to closing the life expectancy gap [3]. 

Aboriginal Health Workers (AHWs) are the first point of contact in the primary health care system for Aboriginal community members. They have a mandate to deliver holistic health care to the broader Aboriginal community [4]. As such, AHWs are central to attempts to influence smoking cessation in the Aboriginal population by providing quit support information, implementing brief tobacco interventions for clients, acting as health advocates, and making referrals [4]. Furthermore, as respected health professionals from whom community members seek advice and guidance, AHWs are well-positioned to function as role models. Evidence indicates, however, that between 41% and 64% of AHWs smoke [5,6,7,8]. Further evidence suggests that AHWs who smoke may smoke heavily [8] and, compared to non-smokers, may be less likely to integrate brief smoking intervention advice into their consultation as they do not feel comfortable giving advice that they themselves do not follow [6].

Smoking among the Aboriginal and Torres Strait Islander population has previously been linked with socio-economic disadvantage [9,10]. Living conditions have also been identified as correlates. Notable examples include living in overcrowded conditions, and renting or living in community housing [11]. In isolation or collectively, these factors may function to encourage the uptake and maintenance of this behaviour [12].

There is limited quantitative evidence regarding AHWs’ readiness to quit smoking or their quit smoking behaviour [13]. Further to this, little such research has addressed the psychosocial, socio-demographic and household smoking factors associated with smoking among this population. Qualitative research provides some insight into the role of these factors. Sharing or smoking cigarettes with family, friends and co-workers has been found to strengthen relationships, to foster a sense of belonging and social cohesiveness [12,14], and, through associated yarning, to enable a forum within which to share feelings and experiences [15]. AHWs often talk to clients over a smoke as it creates a relaxed and comfortable atmosphere for clients to discuss their issues [16]. This can reinforce AHW-client relationships and facilitate yarning that may be important in other areas of their client’s lives. These practices coincide with key cessation barriers identified by AHWs, including lack of support, having a partner who smokes and a “smoky environment” [6]. Life and work-related stress have also been implicated in the maintenance of smoking [6,13,17].

To date no empirical research has sought to reach AHWs at a state or territory level within Australia and contextualise smoking by characterising the psychosocial, socio-demographic and household smoking characteristics associated with smoking status and quit smoking behaviours. Working in collaboration with the Aboriginal Health Council of South Australia (AHCSA), the South Australian Department of Health (SA DoH) sought to develop culturally appropriate cessation strategies for AHWs. As one step in this journey, this study aimed to: (1) characterise the smoking status and quit smoking behaviour of AHWs; and (2) identify the psychosocial, socio-demographic, and household smoking characteristics that distinguish smokers from quitters and never smokers.

## 2. Methods

This study was part of a larger project funded by the SA DoH’s Strategic Health Research Program (SHRP). This research project represented a partnership between the University of South Australia and AHCSA. It took a participatory research approach with university-based researchers sharing decision making with Aboriginal partners in all phases of the research [18,19]. The presence of mutual trust and respect among partners was important to support capacity building, empowerment and ownership and to ensure that culture was respected in the research process [20]. Ethical approval was obtained from the AHCSA Aboriginal Health Research Ethics Committee, the University of South Australia’s Human Research Ethics Committee, and the SA DoH Human Research Ethics Committee.

### 2.1. Participants

This study aimed to reach all Aboriginal people employed as AHWs in SA. The Aboriginal Primary Health Care Workers Forum (APHCWF), comprised of 17 members, represents AHWs in all organisations, health centres and hospitals throughout SA. AHWs hold a minimum Level III Primary Health Care Certificate. A list of AHWs in SA (*n* = 135) was obtained through the AHCSA and updated by contacting APHCWF member organisations. AHWs were eligible to participate in this study if they were: (a) employed in health services, organisations or centres across SA (government and Aboriginal Community Controlled) in either a full or part-time capacity; (b) of Aboriginal or Torres Strait Islander descent; (c) represented by the APHCWF, and (d) working at the time of data collection (*i.e.*, not on extended leave).

### 2.2. Procedures

With the support of the APHCWF, the project was promoted to regional coordinators and Chief Executive Officers (CEOs) of Aboriginal Community Controlled and government health services. Regional coordinators and CEOs were encouraged to allow AHWs work time to complete the survey. Soon after the research project was funded, a SA state-wide Tobacco Coordinator was appointed at AHCSA. The state-wide Tobacco Coordinator, a respected elder, championed the research project and travelled with research team members to visit Aboriginal Health Services. During the visits, the team explained the project and invited AHWs to participate in the study. Surveys were administered on site by researchers in the presence of the state-wide Tobacco Coordinator; some AHWs opted to complete the surveys later in their own time and to return them by mail. Researchers were present to answer questions and provide assistance, if needed. As it was not possible to visit all health services, some surveys were mailed to AHWs.

### 2.3. Instrument

AHWs were administered a 25-minute self-report survey. The survey asked AHWs to complete questions related to their: (a) smoking status and quit smoking behaviour; (b) socio-demographic or living situation, including household exposure to smoking; and (c) psychosocial health. Survey measures were selected for their demonstrated reliability and validity and, where possible, prior use in Aboriginal populations. The survey was checked for readability, relevance and cultural appropriateness by the Aboriginal Steering Committee of the Ministerial Reference Group on Tobacco (ASC-MRGOT). Internal consistency for the psychosocial scales was assessed and found acceptable (α range = 0.69–0.90).

### 2.4. Smoking Status and Quit Smoking Behaviour

To determine smoking status, AHWs were asked if they currently smoked (current smoker), did not currently smoke but had in the past (quitters); or never smoked (never smoker). Smoking status was assessed on a nominal scale.

*Stages of Change* questions assessed smokers’ readiness to quit [21]. Current smokers were asked how many times they had made a quit attempt lasting for at least 24 hours in the previous 12 months and whether they were seriously thinking about quitting smoking. Based on their responses, current smokers were assigned to either pre-contemplation, contemplation, or preparation stages of change. Quitters were asked how long ago they quit smoking and assigned to either the action or maintenance stages of change. In the pre-contemplation stage, a smoker would not be considering quitting within the coming six months. Smokers were assigned to the contemplation stage if they either wanted to quit within the next 30 days, but had not made a quit attempt in the last 12 months, or wanted to quit smoking in the next six months. Smokers in the preparation stage identified that they wanted to quit smoking in the next 30 days and that they had made at least one quit attempt in the last 12 months. Quitters were classified as being in the action stage if they had quit and sustained not smoking within the six months prior to the survey; they were classified as being in the maintenance stage if they had sustained a quit attempt for longer than six months [22]. Stages of change was assessed on a nominal scale.

*Reasons for Quitting*. Quitters were asked to identify their main reason for quitting smoking. Close-ended response options included: doctor’s advice, health concerns, expenses, peer or family pressure. They could also specify another reason by providing an open-ended response. These responses were content analysed by two researchers and disagreements resolved through discussion.

*Quit Supports*. Quitters were asked whether they used nicotine replacement therapy (NRT) to help them quit smoking and, if they had, whether they found NRT had helped them quit smoking or not. To determine if quitters had support to quit or if they had quit on their own (*i.e.*, cold turkey), they were asked to check one of the two following responses: “with outside help” or “on my own”. If they quit “with outside help”, they were asked to identify, in an open-ended response, how they quit. 

*Nicotine dependence* of current smokers was assessed via the eight-item Fagerstrom Tolerance Questionnaire (FTQ) (e.g., number of cigarettes smoked in a day, how soon after you awake do you smoke your first cigarette) for which a maximum of 11 was scored [23]. In addition, participants reported their age at smoking onset, with quitters also asked to report the age they ceased smoking.

### 2.5. Socio-Demographic and Household Smoking Characteristics

AHWs reported their gender, date of birth, marital status (married or de facto relationship *vs.* single), highest level of education achieved (AHW certificate *vs.* higher than AHW certificate), employment status (part-time *vs.* full time), the number of people and children living in their household, and the number of people they supported on their wage. Geographic location was categorised using the AIHW Rural, Remote and Metropolitan Areas classification [24]. The survey also enquired into the following two household smoking characteristics: whether another smoker lived within their household, and whether smoking was permitted inside the house [25]. 

### 2.6. Psychosocial Health

*Stressors* perceived by AHWs were measured using the Life Events Scale [26]. Participants checked any of the 19 items listed that had worried them, their family or close friends during the last year (e.g., serious illness, gambling problem, discrimination/racism). Items were expressed as the number of stressors experienced (α = 0.86). This scale has previously been utilised with Indigenous Australians [27].

*Perceived Social Support* was measured on an eight-item scale [28]. Participants rated how often eight different supports were available to them (e.g., “Someone you can count on when you need advice”) on a four-point Likert scale ranging from “all of the time” to “almost none of the time”. Summed scale scores could range from 8–32, with 8–16, 17–24 and 25–32 indicating a low, medium and high level of social support, respectively (α = 0.90). This measure has previously been utilised with Aboriginal populations in Canada [29].

*Perceived Self-Efficacy* to employ one’s skills to resist temptations to smoke was assessed using Velicer’s [30] nine-item scale. Current smokers identified how tempted they were to smoke in nine different situations such as “with friends at a party” along a five-point Likert scale ranging from 1 (not at all tempted) to 5 (extremely tempted). Self-efficacy was divided into three subscales of positive situations, craving situations and negative situations; individual sub-scale scores were summed and averaged across these three categories to represent a continuous measure (α = 0.84) [30].

*Mastery*, defined as the extent to which one perceives control over the factors affecting one’s life, was assessed using Pearlin’s [31] seven-item scale. Participants rated their level of agreement for the seven items (e.g., “I often feel helpless in dealing with the problems of life”) using a five-point Likert scale ranging from “strongly disagree” to “strongly agree”. Items were summed to represent a continuous measure (α = 0.69). This scale has previously been utilised with Indigenous Australians [32].

*Emotional Wellbeing* was assessed using the Affect Balance Scale [33]. Participants reported if they had experienced in the past few weeks five positive and five negative affective states. Overall affect balance was calculated by subtracting the total negative score from the total positive score and adding a constant of five. Scores could range from zero to 10. A score of 10 indicated the greatest level of affect balance and highest level of emotional wellbeing (α = 0.76). This measure has previously been reported in relation to smoking among Aboriginal people in Canada [34].

### 2.7. Statistical Analysis

Where appropriate, handling of missing data within items on the psychosocial scales was managed with person mean substitution [35]. Continuous measures were reported using median and inter-quartile range (IQR) and categorical measures as proportions. Prior to the application of formal analyses, the normality of variables was assessed with the Shapiro-Wilk test. As a majority of variables violated assumptions of normality, it was necessary to apply non-parametric statistics for analyses. Of these, the Kruskal-Wallis test was used to test differences in continuous variables between never smokers, quitters and current smokers. If the Kruskal-Wallis test was statistically significant the Mann-Whitney U-test was employed as a post-hoc test to evaluate differences in specific contrasts between pairs of smoking status groups. For ordinal and nominal level variables, group differences in smoking status were assessed via the Chi-square test of independence. If the overall Chi-square test was significant, further Chi-square analyses were conducted between pairs of smoking status groups (post-hoc). Data were analysed using SPSS for Windows, version 17 [36]. Consistent with previous studies [37,38], p-values of 0.05–0.10 were considered to represent borderline levels of statistical significance for all tests due to the study’s small sample size, and non-parametric tests having low statistical power.

## 3. Results

### 3.1. Participant Characteristics

Eighty-five surveys were completed for a response rate of 63.0% (*n* = 135). Participants ranged in age from 21–67 years with a median age (IQR) of 42 (33, 48) years. The gender representation within the sample (66% female) did not differ from the gender composition of AHWs working in SA at the time of the study (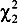 = 0.43, *p* = 0.51). A greater proportion of respondents worked in rural health services (51.8%) as compared to metropolitan (28.2%) or remote health services (20.0 %), with this distribution reflective of the underlying geographic distribution of AHWs (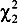 = 0.57, *p* = 0.75). The median (IQR) number of people living within an AHW’s household was 3 (2, 4), with the median (IQR) number of resident children being 1 (0, 2). The majority of AHWs were employed full-time (91.6%), with the remainder employed on a part-time basis. AHWs supported approximately two people (IQR = 1, 4) on their wage.

### 3.2. Smoking Status, Socio-Demographic, Household Smoking and Psychosocial Characteristics

The prevalence of cigarette smoking across smoking status groups was 50.6% (*n* = 43 “current smokers”). Of AHWs responding, 22.4% reported having smoked in the past, but not currently (*n* = 19, “quitters”) and 27.1% reported never having smoked (*n* = 23 “never smokers”). Although smoking status did not differ by gender (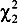 = 2.71, *p* = 0.26), among women 55.4% self-identified as current smokers compared to 41.4% of men. From a geographic perspective, crude smoking prevalence ratios were 62.5% in metropolitan regions, 45.5% in rural localities and 47.1% in remote areas. No differences in smoking status were found across geographic locations (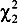 = 2.34, *p* = 0.67).

In comparing socio-demographic and household smoking characteristics across smoking status groups (Table 1 and Table 2), differences were found for living with a smoker (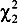 = 22.28, *p* < 0.001) and employment status (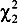 = 5.66, *p* = 0.06). Gender (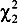 = 2.72, *p* = 0.26), age (*H* = 1.84, *p* = 0.40), number of children living with the AHW (*H* = 0.67, *p* = 0.72), number of people in the household (*H* = 1.59, *p* = 0.45) and number of people supported on their wage (*H* = 1.48, *p* = 0.48) did not differ by smoking status.

**Table 1:** Socio-demographic variables according to smoking status (median (IQR))^ †^.

	*n*	Never Smoker	Quitter	Current Smoker
Age	81	42.0 (35.0, 47.5)	44.5 (36.5, 48.0)	39.5 (29.8, 48.3)
Number of children in household	82	1.0 (0.0, 2.0)	2.0 (0.0, 2.0)	1.0 (0.0, 2.3)
Household size	81	2.0 (1.0, 4.0)	2.5 (1.8, 3.1)	3.0 (1.5, 4.0)
Supporting on wage	82	2.0 (1.0, 4.0)	2.5 (1.0, 4.1)	1.5 (1.0, 3.0)

^†^ Discrepancy in participant numbers from overall *n* due to missing data unable to be substituted.

**Table 2:** Socio-demographic and household smoking variables according to smoking status (% (*n*)).

	Never Smoker	Quitter	Current Smoker
Gender: Male	47.8 (11)	31.6 (6)	27.9 (12)
Married/de facto relationship	69.6 (16)	83.3 (15)	58.1 (25)
Education: AHW certificate	81.8 (18)	73.7 (14)	90.7 (39)
Employment status: Full time *	95.7 (22)	77.8 (14)	95.2 (40)
Living with a smoker **	25.0 (5)	33.3 (6)	82.1 (32)
Smoking inside the house	20.0 (3)	5.9 (1)	30.0 (12)

** significant difference observed between smoking status groups using the Chi-square test (*p* < 0.05);* borderline difference observed between smoking status groups using the Chi-square test (*p* < 0.10).

Psychosocial factors were compared across smoking status groups (Table 3). A statistically significant difference in emotional wellbeing was observed across smoking status groups (*H* = 7.08, *p* = 0.03). Perceived social support varied according to smoking status (*H* = 5.79, *p* = 0.06), however stressors (*H* = 0.30, *p* = 0.86) and mastery (*H* = 1.52, *p* = 0.47) did not.

**Table 3:** Psychosocial variables according to smoking status (median (IQR))^ †^.

	*n*	Never Smoker	Quitter	Current Smoker
Stressors	82	3.0 (2.0, 8.0)	3.0 (1.0, 5.0)	3.5 (2.0, 6.8)
Mastery	82	25.0 (22.0, 29.0)	26.0 (24.0, 29.5)	26.0 (22.0, 28.0)
Social support *	81	23.0 (18.0, 28.0)	26.3 (22.3, 30.0)	22.0 (20.0, 26.0)
Affect balance **	82	8.0 (5.0, 9.0)	7.0 (5.0, 9.0)	5.0 (8.0, 4.0)

^†^ Discrepancy in participant numbers from overall n due to missing data unable to be substituted; ** significant difference observed between smoking status groups using the Kruskal-Wallis test (*p* < 0.05); * borderline difference observed between smoking status groups using the Kruskal Wallis test (*p* < 0.10).

To pinpoint the nature of the differences between the three smoking status groups, post-hoc comparisons were conducted for variables in which statistically significant or borderline group differences were found. Post-hoc comparison results and descriptive information for current smokers and quitters are reported below.

### 3.3. Current Smokers

Current smokers reported smoking for a median (IQR) of 23 (13.5, 30.5) years. Daily consumption rates varied widely within this group, with 69.0% (*n* = 42) smoking between 1 and 15 cigarettes per day, 19.0% between 16 and 25 cigarettes and 11.9% more than 26 cigarettes. Median (IQR) nicotine dependence and perceived self-efficacy scores for current smokers was 5 (4, 8) and 11 (10, 13) respectively. In the 12 months prior to survey completion, 50.0% of current smokers reportedly had made at least one quit attempt. At survey completion 69% of current smokers demonstrated a readiness to quit smoking with 61.9% in the contemplation stage, and 7.1% in the preparation stage. The remaining 31.0% of current smokers were in the pre-contemplation stage. 

*Post-hoc* comparisons revealed current smokers were more likely to live with another smoker than never smokers (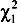 = 18.40, *p* < 0.01) and quitters (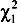 = 13.15, *p* < 0.01). Emotional well-being as measured through the Affect Balance Scale was lower for smokers than for both quitters (*U* = 230.5, *p* = 0.03) and never smokers (*U* = 325.5, *p* = 0.03).

### 3.4. Quitters

Of quitters, 22.2% were in the action stage and 77.8% were in the maintenance stage. The median (IQR) ages at which quitters started and stopped smoking were 16 (14, 17) and 36 (22, 46) years, respectively. Prior to quitting, 71.4% of quitters had made at least one quit attempt that lasted for a week or more. A majority of quitters (88.2%) reported they had quit smoking on their own, without outside help. Of AHWs who quit smoking, only one had used NRT; this person indicated that it had not helped them quit smoking. Fifty-percent of quitters identified “health concerns” as the primary impetus for quitting smoking. Less frequently reported reasons for quitting smoking included smoking was “not their thing”, expenses, pregnancy, family death, role model and shame. 

Results of post-hoc comparisons indicated that a greater proportion of quitters, compared to current smokers, reported working part-time (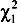 = 4.27, *p* = 0.04) with a borderline difference between quitters and never smokers (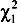 = 3.01, p=0.08). Quitters also reported higher levels of social support than never smokers (*U* = 131.5, *p* = 0.04) and current smokers (*U* = 243, *p* = 0.03). Quitters did not differ from current smokers in terms of age of smoking onset (*U* = 328, *p* = 0.63). 

## 4. Discussion

Our survey findings demonstrate that approximately one-half (50.6%) of AHWs within the state of South Australia currently smoke. This prevalence is consistent with previous AHW studies in Australia [5,6,7,8] and indicates that this cohort of AHWs smoke at a similar rate to the broader state-situated Aboriginal population [1], and more than double that of the total population of South Australia [39]. 

Beyond prevalence, this is the first study to quantitatively assess the quit readiness of AHWs at the state-level in Australia. Observations demonstrate a substantive desire to quit smoking in the AHW workforce with 69% of current smokers indicating a level of readiness to quit while 50% had attempted to do so in the preceding twelve-month period. Previous AHW studies have reported similar observations [6,7], however these studies did not apply the stages of change model utilised by this study. Our results are in line with recent national-level estimates from the general Aboriginal and Torres Strait Islander population, which highlight two-thirds of smokers to have attempted to quit or reduce smoking in a preceding twelve-month period [1]. However, some caution is required in exercising such direct comparisons, as national estimates combine data from individuals who had attempted to quit as well as from those who had sought to cut down on their cigarette consumption [1]. 

Study findings additionally highlight important ‘contextual’ factors influencing the smoking behaviour of AHWs. Key amongst these is the high prevalence rate, which functions to normalise smoking in this population. Compounding this, of AHWs who were current smokers, 82% lived with another current smoker. Not only is this figure greater than the current national average for the general Aboriginal and Torres Strait Islander population [1], it contrasts with the proportions of never smokers (25%) and quitters (33%) who reported living with a smoker. 

In addition to greater social exposure to smoking, current smokers reported lower levels of perceived social support compared to quitters. Ceding that such a difference was not evident between current smokers and never smokers, it may be that AHWs who have quit smoking have people around them that care, they can count on, talk to about their problems, spend time with and go to for advice. This concept may be worthy of a qualitative study to further investigate individual differences between quitter and never-smokers. Given the great demands placed on AHWs, in addition to their poor remuneration and a general undervaluing of their role [6,7], it is plausible that AHWs smoke to manage the stress of their position—the burden being greater for those workers without people around to provide instrumental and emotional support. This also would make it more difficult for AHWs who smoke to either attempt to, or successfully quit smoking. Consistent with these observations, we also noted that a greater proportion of quitters worked on a part-time basis. Here, an inference could potentially be drawn between work-related stress and smoking as stress relief [8].

Whilst stress has previously been linked to smoking among AHWs [6,7,8], and indeed herein has been signified as a factor of relevance, perceived stressors failed to differentiate between smoking status. An explanation of this observation may lie in the nature of the stressors scale employed, which broadly focused on ‘life stressors’. Further research should aim to investigate the explicit role of work-related stress and work-life balance through the use of a more work-oriented scale.

Another factor, which contrary to expectations [34] also failed to distinguish between members of smoking classes, was mastery. It is plausible that the absence of an association arose as an artefact of the sample size.

Finally, it is a concern that in addition to experiencing lower levels of social support compared to never smokers, current smokers reported lower levels of emotional well-being by experiencing more negative feelings including loneliness, depression and, or unhappiness. The finding of this study is consistent with previous research [34].

### 4.1. Strengths, Limitations and Challenges

The state-wide focus combined with participatory approach to the design, collection, analysis and interpretation of the findings are two key study strengths. This project demonstrated the utility of a strong partnership between an Aboriginal Community Controlled organisation and researchers embedded in a traditional university setting. Moreover, the up-front adoption of a participatory research approach enabled Aboriginal stakeholders and academic researchers to engage in a two-way learning process where each partner benefitted from the other’s expertise. Not only did this aspect enhance the design and collection process of the survey, it afforded greater insight into the meaning of study findings and their implications for cessation. 

Identification of the AHW workforce is a challenging task due to high AHW employment turnover coupled with ongoing changes to the organisation of Aboriginal health services within the state. These factors have previously been highlighted as impeding research involving AHWs [5]. To overcome this difficulty, repeated contact with health services was made to attain up-to-date lists of AHWs. The achieved response rate was 63%, which is comparable to the few surveys that have explored smoking among AHWs in the Australian context (64.3% [5], 79.0% [6] and 53.6% [7]), but lower than the state-wide response rate (78%) to the most recent National Aboriginal and Torres Strait Islander Social Survey (NATSISS 2008) [1]. This combined with the size of the AHW workforce resulted in a relatively small sample. From a substantive perspective, whilst we were able to derive important insights from attained data, this factor hindered the ability to detect statistically significant differences between smoking status groups. Given the difficulties in defining the AHW workforce, it is difficult to determine the degree to which our sample is representative of the AHW population. It is however relevant to note that in so far as geographic locality and gender are concerned; responses were proportionate to the distribution of these characteristics in the AHW population working in SA Health Services during the study period.

### 4.2. Implications for Smoking Cessation Efforts

AHWs play a pivotal role in the delivery of smoking cessation to their clients but reportedly are less able to deliver these services when they themselves are current smokers [6]. AHWs have thus been recognised as a priority group for state-wide smoking cessation efforts. The continued identification of a high prevalence of smoking among AHWs reinforces the need to encourage smoking cessation and to provide further support to maintain cessation amongst those who have successfully quit.

Based on our findings on the psychosocial, socio-demographic and household smoking factors associated with smoking status, we recommend the application of an ecological approach [40] to support smoking cessation in AHWs. Whilst it may be important to provide AHWs with the skills and knowledge to quit smoking, including inducements regarding health benefits, cessation efforts should not be restricted to the individual level. Given the history of sustained exploitation and negative impacts of colonisation that has shaped and contributed to the normalisation of smoking in Aboriginal Australians [41], every effort must be made to address the social determinants that influence smoking and the environment in which AHWs function among AHWs. Such effort aligns with ecological health promotion principles. These emphasise multi-level, multi-targeted, environmental changes to enable and reinforce AHW’s efforts to quit smoking, which, consistent with social determinants of health frameworks, would lead to improvements in quality of life and health status [42,43]. It is of interest that NRT did not feature prominently in the cessation attempts made by AHWs. Given that NRT is effective in increasing the likelihood of cessation success [41], it would be worthwhile to further explore why AHWs who quit smoking did not use NRT.

Finally, health-concerns were a powerful motivator leading to successful cessation. Previous research on health beliefs and glycaemic control in Aboriginal people with diabetes confirms that concern about severe health outcomes is a powerful predictor of improvements in glycaemic control [44]. Health professionals could use adult health checks as an opportunity to motivate AHWs to quit smoking, reinforcing the dangers of smoking and the benefits that accrue after quitting.

## 5. Conclusions

With 50.6% of AHWs in SA currently smoking and 69.0% demonstrating a readiness to quit, preventative action is required to decrease the rate of smoking in this population. Significantly, smoking status varied by emotional wellbeing, employment status, perceived social support, and living with another smoker. Health concern emerged as a powerful motivator leading to successful cessation. Based on our findings we recommend the application of an ecological approach to support smoking cessation in AHWs. 
